# Short-Term Sustained Hypoxia Elevates Basal and Hypoxia-Induced Ventilation but Not the Carotid Body Chemoreceptor Activity in Rats

**DOI:** 10.3389/fphys.2018.00134

**Published:** 2018-02-27

**Authors:** Karine C. Flor, Elaine F. Silva, Miguel F. Menezes, Gustavo R. Pedrino, Eduardo Colombari, Daniel B. Zoccal

**Affiliations:** ^1^Department of Physiology and Pathology, São Paulo State University, Araraquara, Brazil; ^2^Department of Physiological Sciences, Federal University of Goiás, Goiânia, Brazil

**Keywords:** active expiration, carotid body, chemoreceptor, hypoxic ventilatory response, sympathetic activity, ventilatory acclimatization

## Abstract

Exposure to chronic sustained hypoxia (SH), as experienced in high altitudes, elicits an increase in ventilation, named ventilatory acclimatization to hypoxia (VAH). We previously showed that rats exposed to short-term (24 h) SH exhibit enhanced abdominal expiratory motor activity at rest, accompanied by augmented baseline sympathetic vasoconstrictor activity. In the present study, we investigated whether the respiratory and sympathetic changes elicited by short-term SH are accompanied by carotid body chemoreceptor sensitization. Juvenile male Holtzman rats (60–80 g) were exposed to SH (10% O_2_ for 24 h) or normoxia (control) to examine basal and hypoxic-induced ventilatory parameters in unanesthetized conditions, as well as the sensory response of carotid body chemoreceptors in artificially perfused *in situ* preparations. Under resting conditions (normoxia/normocapnia), SH rats (*n* = 12) exhibited higher baseline respiratory frequency, tidal volume, and minute ventilation compared to controls (*n* = 11, *P* < 0.05). SH group also showed greater hypoxia ventilatory response than control group (*P* < 0.05). The *in situ* preparations of SH rats (*n* = 8) exhibited augmented baseline expiratory and sympathetic activities under normocapnia, with additional bursts in abdominal and thoracic sympathetic nerves during late expiratory phase that were not seen in controls (*n* = 8, *P* < 0.05). Interestingly, basal and potassium cyanide-induced afferent activity of carotid sinus nerve (CSN) was similar between SH and control rats. Our findings indicate that the maintenance of elevated resting ventilation, baseline sympathetic overactivity, and enhanced ventilatory responses to hypoxia in rats exposed to 24 h of SH are not dependent on increased basal and sensorial activity of carotid body chemoreceptors.

## Introduction

In mammals, a prompt increase in pulmonary ventilation emerges at the onset of hypoxia due to the reduction of arterial partial pressure of oxygen (PO_2_) and the consequent stimulation of peripheral chemosensitive cells, mainly located in the carotid bodies (Lahiri et al., 2006). This ventilatory response to acute hypoxia is supported by increases in inspiratory and expiratory motor activities that elevate respiratory frequency and tidal volume (Mateika et al., 1996; Lemes and Zoccal, 2014). Hypoxia also promotes an increase in sympathetic outflow, which adjusts cardiac output and vascular conductance to assure O_2_ tissue perfusion to critical organs (Braga et al., 2007a,b; Hainsworth et al., 2007), as well as elicits reflex metabolic adjustments, including a decrease in body temperature (Madden and Morrison, 2005; Mortola, 2016) and reductions in oxygen consumption (Mortola and Matsuoka, 1993) and ATP demand (Hochachka et al., 1996). Upon reoxygenation, carotid body sensory activity as well as the coupled cardiorespiratory and metabolic responses are progressively restored with the normalization of arterial O_2_ levels. Under conditions of chronic exposure to sustained hypoxia (SH), as experienced at high altitudes, resting ventilation progressive increases, a phenomenon named as ventilatory acclimatization to hypoxia (VAH) (Powell et al., 2000). The magnitude of VAH is time-dependent (hours to years) and may persist upon the return to normoxic conditions (Powell et al., 1998).

Functional plasticity in the peripheral chemoreceptors and brainstem regions processing the chemoreceptor afferent inputs are proposed as a major mechanism underpinning the VAH (Smith et al., 1986; Dwinell and Powell, 1999). VAH is also accompanied by amplified ventilatory responses to a new challenge of hypoxia (Barnard et al., 1987; Powell et al., 1998), indicating a sensitization of peripheral chemoreflex pathway. It is suggested that carotid body chemosensory sensitization occurs during the early stages of exposure to chronic SH, while modification in the gain of the central mechanisms required longer periods of exposure (Bisgard, 2000; Wilkinson et al., 2010). In goats, carotid body chemoreceptor afferent activity enhances after 6 h of chronic SH (Powell et al., 1998) whilst, in cats, this adaptation was reported to occur after 48 h of exposure (Vizek et al., 1987). In a recent study, we demonstrated that rats exposed to 24 h of chronic SH exhibit persistent changes in their breathing pattern (Moraes et al., 2014). Using a decerebrated *in situ* rat preparation, we verified that chronic SH exposure for 24 h generates the pattern of active expiration at resting conditions, with the emergence of high-amplitude bursts in abdominal motor activity and reduced upper airway resistance (Moraes et al., 2014). However, there is no evidence indicating that the changes induced by 24 h of chronic SH in the respiratory motor activity reported *in situ* are associated with modifications in pulmonary ventilation measured *in vivo*. Also it remains to be elucidated whether the emergence of active expiration in rats exposed to this short-term paradigm of SH is associated with augmented carotid body chemosensory afferent activity.

In association with the changes in the respiratory pattern, rats exposed to 24 h of chronic SH present a sustained increase in baseline sympathetic activity and arterial pressure levels (Moraes et al., 2014). The augmented sympathetic activity after short-term SH was entrained with the emergence of abdominal expiratory hyperactivity, suggesting a common etiological mechanism. We previously identified that 24 h of chronic SH causes a long-lasting activation of expiratory neurons of the parafacial respiratory group (pFRG) (Moraes et al., 2014). The pFRG is located at the ventral surface of medulla, ventral to the facial nucleus, and is essential for the generation of active expiratory pattern (Janczewski and Feldman, 2006). Neurons of the pFRG provides excitatory inputs to bulbospinal pre-motor expiratory neurons of the ventral respiratory column in conditions of elevated respiratory drive (Silva et al., 2016), generating the expiratory bursts in abdominal activity. The pFRG is also suggested to send excitatory drive to pre-sympathetic neurons of the ventral medulla (Molkov et al., 2011; Moraes et al., 2014), contributing to expiratory-related bursts in sympathetic outflow during metabolic challenges, such as hypoxia. The pFRG neurons are activated when peripheral chemoreceptors are stimulated (Moraes et al., 2012), allowing us to speculate that 24 h of chronic SH may enhance the afferent activity of carotid body chemoreceptors and then contribute to stimulate the pFRG neurons under resting conditions.

Based upon these observations, in the present study we considered the hypothesis that the maintenance of elevated abdominal expiratory motor output and sympathetic activities of rats exposed to chronic SH for 24 h are mainly dependent on baseline and sensory afferent hyperactivity of carotid body peripheral chemoreceptors. To explore this possibility, we examined pulmonary ventilation and body temperature of unanesthetized, SH-conditioned rats under normoxic/normocapnic conditions and during a new challenge of hypoxia. We also evaluated basal and hypoxic-induced afferent activity of carotid sinus nerve (CSN) of *in situ* preparations of rats previously exposed to SH for 24 h.

## Material and methods

### Animals and ethical approval

Experiments were performed on male Holtzman rats weighing 60–80 g (23–28 days old), randomly divided in two experimental groups: rats exposed to short-term SH (SH, 10% O_2_ for 24 h) and rats maintained under normoxic conditions (control, 21% O_2_ for 24 h). The rats were obtained from the Animal Care Unit of the São Paulo State University, Araraquara, and housed with free access to rat chow and water, under controlled conditions of temperature (22 ± 1°C), humidity (50–60%), and light/dark cycle (12:12 lights on at 07:00 a.m.). All experimental procedures comply with the Guide for the Care and Use of Laboratory Animals published by the Brazilian National Council for Animal Experimentation Control (CONCEA), and were approved by the Local Ethical Committee in Animal Experimentation of School of Dentistry of Araraquara, São Paulo State University (protocols 18/2014 and 7/2015).

### Sustained hypoxia

Animals of both groups were housed in collective cages placed inside chambers (volume: 100 L) that allowed the control of fraction of the inspired oxygen (FiO_2_). The conditions of the temperature, humidity, light/dark cycle, and food/water free access were kept as aforementioned. In the chamber of SH group, injections of N_2_ (100%) were performed to reduce FiO_2_ from 21 to 10%, remaining at this level for 24 h. The control group received short-term injections of O_2_ and N_2_ to maintain FiO_2_ at 21% for 24 h. The control of O_2_ and N_2_ injections (White Martins, Sertãozinho, Brazil) in the chambers was performed through a system of solenoid valves controlled automatically by a computerized system (Oxycycler, Biospherix, Lacona, NY, USA). O_2_ levels were continuously monitored through sensors located inside the chamber. Gas injections were performed in the upper portion of the chambers to prevent air jets from directly reaching the animals. At the end of the experimental protocol, the animals were removed from the chambers and then conducted for the experiments.

### Evaluation of the body temperature

Two days before the exposure to SH or normoxia, the animals were anesthetized with halothane (Tanohalo, Cristália, SP) and a small incision (1–1.5 cm) was made in the abdomen to gently implant the temperature-monitoring probes (SubCue Datalogger Standard, Alberta, Canada) into the abdominal region of the animal. After the incision suture, the animals received the veterinary pentabiotic (penicillin–streptomycin, 1,200,000 IU, 0.1 ml/100 g, i.p.) and the anti-inflammatory ketoprofen (1%, 0.03 ml/100 g, s.c.), and were monitored until regain consciousness. The temperature probes were programmed to continuously record body temperature of the animals, every 2 min, from 4 h before the initiation of SH/normoxia protocol to the end of experiments. After, the animals were sacrificed with anesthetic overload (pentobarbital, 0.4 ml/100 g, i.p.), the probes were removed from the abdominal cavity and the data were transferred to a computer using appropriate software for analysis (The SubCue Analyzer, Alberta, Canada).

### Evaluation of the respiratory parameters *in vivo* during normoxia and hypoxia

Pulmonary ventilation was measured in unrestrained animals by whole-body plethysmography (Emka Technologies, Paris, France). Animals from SH and control groups were individually kept into a small chamber (5 L) continuously flushed with humidified room-temperature air (delivered at 1.5 L.min^−1^). This is an open system, so the respiratory signal reflects an indirect measurement of airflow from the pressure variations generated by the inspiratory and expiratory flows. The respiratory-related pressure fluctuations were amplified (X 500) and acquired at 1 kHz (IOX, version 2.8, Emka Technologies). Chamber temperature, relative humidity, and atmospheric pressure were also monitored throughout the experiments. Immediately after the hypoxia or normoxia protocol, animals were placed inside the chamber and a period of 45–60 min was allowed for animal stabilization and acclimatization. After this period, baseline ventilation was initially monitored for 30 min, followed by a new episode of hypoxia (7% of O_2_ balanced with N_2_, 1L.min^−1^) for 20 min and a period of recovery (return to normoxia) of 40 min. The hypoxic gas was delivered into the chamber using a computer-driven mass flow regulator (Alicat Scientific, Tucson, USA). The parameters evaluated were: (i) respiratory frequency (Rf; cycles per minute), derived from the time interval between consecutive inspiratory flow peaks; (ii) tidal volume (V_T_, ml.100 g^−1^), estimated using the equation of Drorbaugh and Fenn (1955), considering as reference a calibrated air volume injected from a 5-ml syringe; (iii) inspiratory and expiratory times (ms); and (iv) peaks inspiratory and expiratory flows (mL.s^−1^). Analyses were performed during periods of quiet breathing. The responses during hypoxia were evaluated from min 5 to min 20 of exposure and reported as average values over 1 min. Animals' temperature was continuously monitored during baseline, hypoxia and recovery period.

### Recordings of phrenic, abdominal, thoracic sympathetic and carotid sinus nerve activities in the decerebrated, arterially perfused *in situ* preparation

Approximately 30 min after the end of SH or control protocols, the animals were submitted to surgical procedures to obtain the *in situ* preparations, as previously described (Zoccal et al., 2008). Briefly, animals were deeply anesthetized with isofluorane and transected sub-diaphragmatically. The head and thorax were immersed in cooled (2–4°C) Ringer solution (in mM: 125 NaCl; 25 NaHCO_3_; 3.75 KCl; 2.5 CaCl_2_; 1.25 MgSO_4_; 1.25 KH_2_PO_4_, and 9.9 glucose) and the cerebral cortices, hippocampus and thalamic areas were gently removed by aspiration. The preoptic area, the adjacent septal nuclei, and hypothalamic areas remained intact. The preparation was then skinned and transferred to a recording chamber. The descending aorta was isolated for cannulation, followed by removal of the ribs on the right side to access to the paravertebral sympathetic chain (tSN), at the level of T11–T12. The lungs were removed and the left phrenic nerve (PN) was isolated, cutting it at its insertion to the diaphragm. The CSN was isolated close to its insertion into the glossopharyngeal nerve and sectioned. The abdominal nerve (AbN) was dissected from the oblique abdominal muscle at the thoracolumbar level and distally sectioned. Preparations were then transferred to a recording chamber and the descending aorta was cannulated with a double-lumen cannula and retrogradely perfused with modified Ringer solution containing an oncotic agent (Polyethylene glycol 20,000, 1.25%, Sigma-Aldrich, USA) and a neuromuscular transmission blocker (vecuronium bromide 3–4 μg.ml^−1^). The perfusion was performed using a peristaltic pump (Watson-Marlow 502S, USA) and perfusion pressure was maintained between 50 and 70 mmHg through adjustments in the flow (maintained between 21 and 25 mL.min^−1^) and by the addition of vasopressin (0.6–1 nM, Sigma, USA) to the perfusate. The perfusion solution was constantly gassed with 95% O_2_-5% CO_2_ mixture (White Martins, São Carlos, Brazil) for gas supply and maintenance of the pH (7.40), warmed (31–32°C), and filtered using a polypropylene filter (25 μm pore size). A syringe containing potassium cyanide (KCN, 0.05%) was connected to the perfusion system to allow small acute infusions (50 μL) to stimulate peripheral chemoreceptors, as previously described (Moraes et al., 2012). The perfusion pressure was monitored through a pressure transducer (MLT06070, ADInstruments, Bella Vista, Australia) that, in turn, was connected to an amplifier (Grass Quad Amplifier, model 15LT, RI, USA). Recordings of PN, AbN, tSN, and CSN activities were obtained using bipolar suction electrodes connected to 3D micromanipulators. Bioelectric signals were amplified (CP511 Amplifier, Grass Technologies, RI, USA), filtered (0.3–3 kHz), and acquired by A/D converter (micro1401, Cambridge Electronic Design Limited, Cambridge, England) on a computer using Spike 2 software (version 7, Cambridge Electronic Design).

The analyses of the nerve activities were performed on rectified and smoothed signals (time constant of 50 ms) using custom-written scripts in Spike 2 software. PN activity was evaluated by its burst amplitude (μV) and frequency (bursts per minute, bpm). AbN activity was calculated as the mean activity (μV) during the expiratory phase. tSN activity was determined as the mean activity (μV) during inspiratory phase (coincident with PN burst), first stage of expiration (E1, corresponding to the initial 2/3 of expiratory phase) and second stage of expiration (E2, corresponding to the final 1/3 of expiratory phase) (Richter and Smith, 2014). The magnitude of the responses to peripheral chemoreceptor activation (with KCN) were calculated as the variation between the peak response and the immediate baseline activity, and expressed as percentage values (%), with exception of PN burst frequency.

### Data analyses

The results were expressed as mean ± standard error of mean. Baseline parameters *in vivo* and *in situ*, and the hypoxic responses *in situ* were compared using Student's *t*-test. Analyses of ventilatory and body temperature responses to hypoxia *in vivo* were performed using two-way ANOVA repeated measurements, followed by Bonferroni post-test. The confidence level (confidence interval, CI) was set as 95% and the differences were considered statistically significant when *P* < 0.05. Graphic operations and statistical analyses were performed using GraphPad Prism software (version 6, GraphPad, La Jolla, USA).

## Results

### Ventilatory parameters and body temperature after 24 h of SH *in vivo*

**Figure 1:**
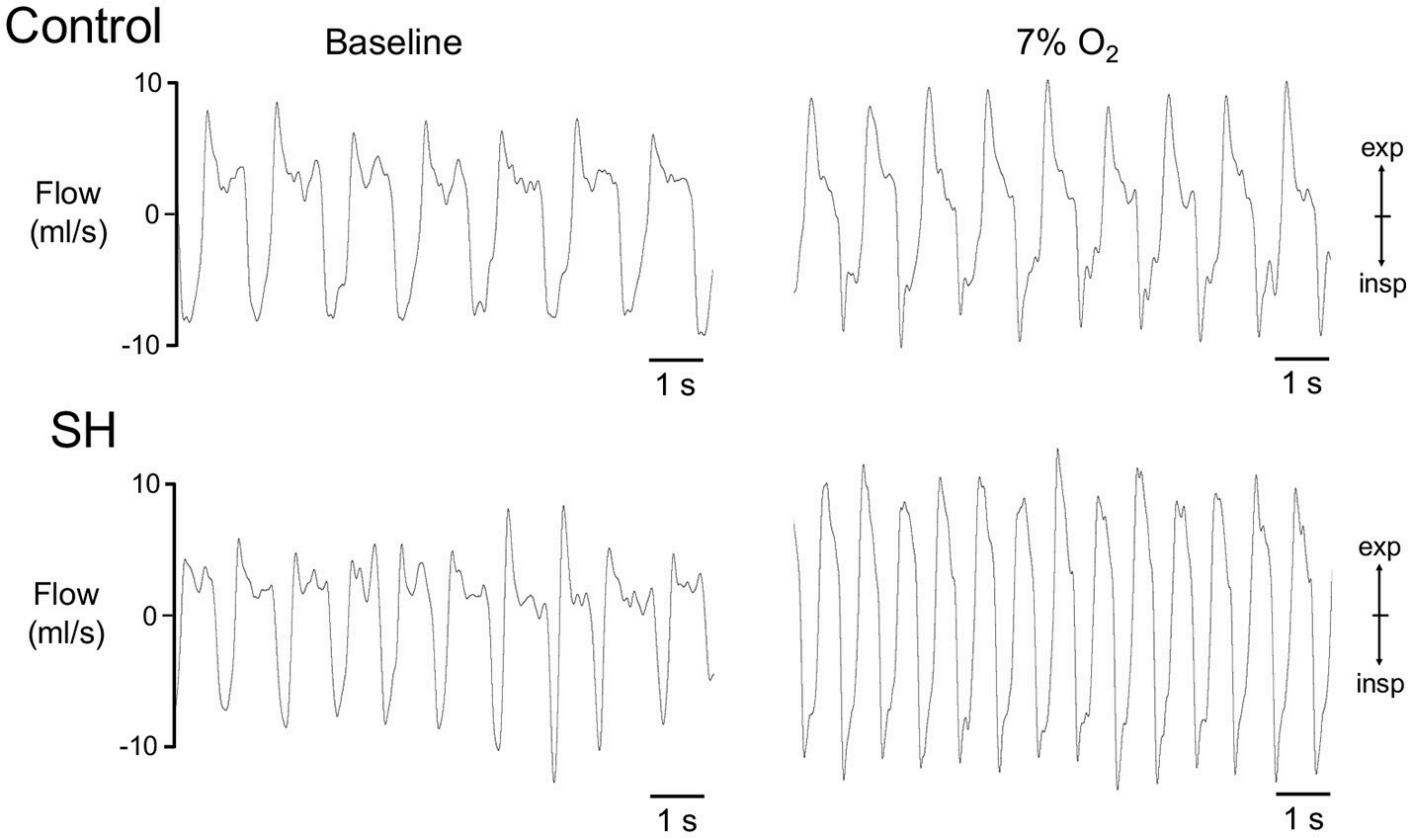
Pulmonary ventilation of control and SH rats. Recordings of airflow of control and SH rats, representative from their respective groups, illustrating the ventilatory pattern during baseline and during a new hypoxic episode (7% O_2_).

**Figure 2:**
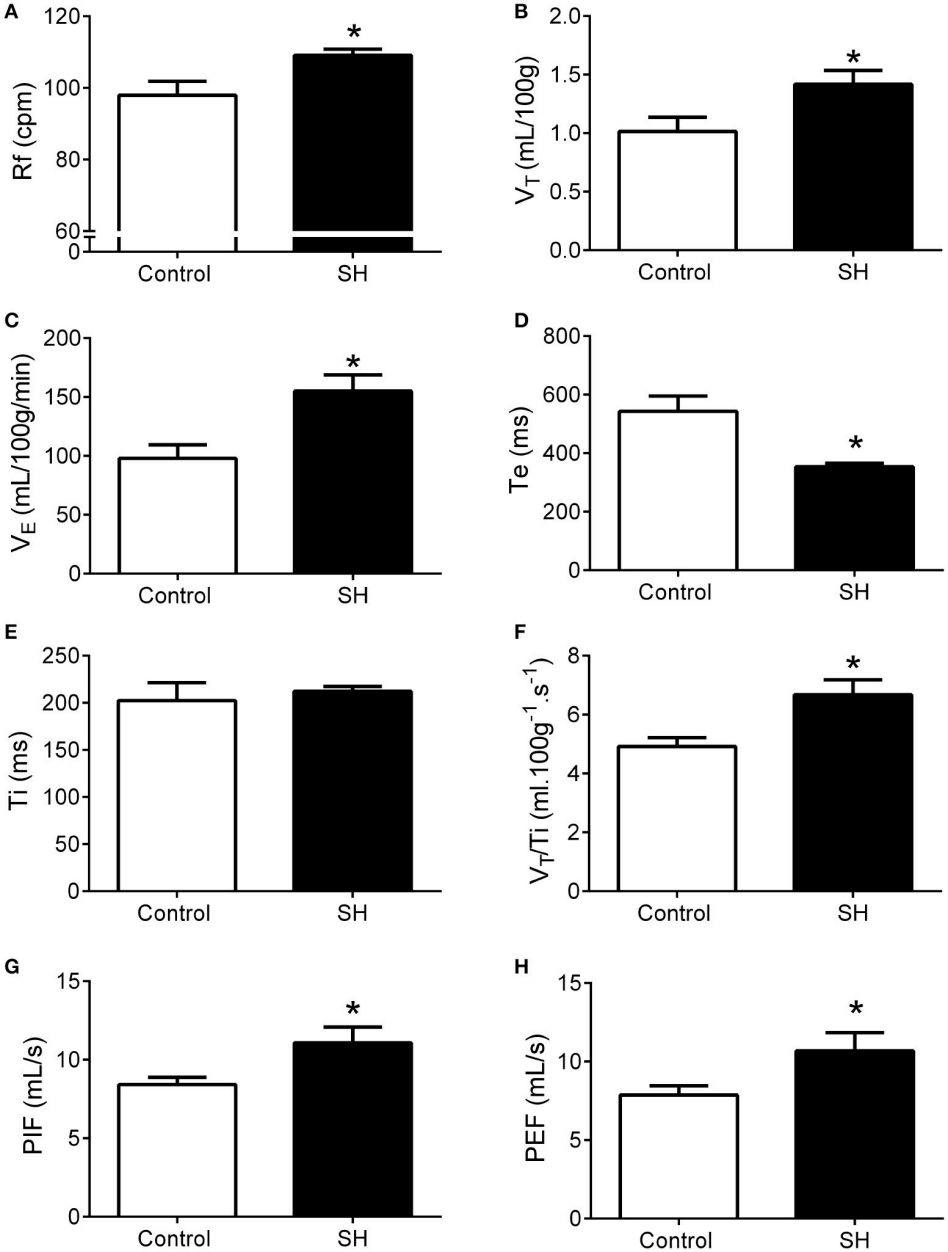
Baseline ventilation in rats after 24 h of sustained hypoxia. Respiratory frequency (Rf, **A**), tidal volume (V_T_, **B**), minute volume (V_E_, **C**), expiratory (Te, **D**), and inspiratory (Ti, **E**) time, tidal volume to inspiratory time ratio (V_T_/T_i_, **F**), peak inspiratory flow (PIF, **G**), and peak expiratory flow (PEF, **H**) of rats exposed to 24 h of sustained hypoxia (SH, *n* = 12) or maintained under normoxia (control, *n* = 11). *Different from control group, *P* < 0.05.

Representative recordings of pulmonary ventilation of control and SH-conditioned rats are illustrated in Figure 1. Under resting conditions (normoxia/normocapnia), rats exposed to short-term SH (*n* = 12) exhibited elevated respiratory frequency (109 ± 2 vs. 98 ± 4 cpm, 95% CI: 105–113 vs. 90–107, *P* = 0.0161; Figure 2A), tidal volume (1.42 ± 0.11 vs. 1.01 ± 0.12 ml. 100 g^−1^, 95% CI: 1.15–1.68 vs. 0.74–1.28, *P* = 0.0277; Figure 2B), and minute ventilation (155.0 ± 13.8 vs. 97.9 ± 11.4 ml. 100 g^−1^.min^−1^, 95% CI: 124.7–185.5 vs. 72.8–123.1, *P* = 0.0042; Figure 2C) compared to control rats (*n* = 11). The changes of SH group were associated with: (i) reduction in the expiratory time (354 ± 12 vs. 521 ± 52 ms, 95% CI: 327–380 vs. 428–658, *P* = 0.0019, Figure 2D), but not in the inspiratory time (212 ± 5 vs. 212 ± 17 ms, 95% CI: 200–223 vs. 160–244, *P* = 0.6206; Figure 2E); (ii) increase in tidal-volume to inspiratory-time ratio (6.7 ± 0.5 vs. 4.9 ± 0.3 ml.100 g^−1^.s^−1^, 95% CI: 5.6–7.8 vs. 4.2–5.6, *P* = 0.0083; Figure 2F); and (iii) increases in peak inspiratory (11.1 ± 1.0 vs. 8.4 ± 0.5 mL.s^−1^, 95% CI: 8.9–13.3 vs. 7.4–9.4, *P* = 0.0254; Figure 2G) and expiratory flows (10.7 ± 1.2 vs. 7.9 ± 0.6 mL.s^−1^, 95% CI: 8.1–13.2 vs. 6.6–9.15, *P* = 0.0426; Figure 2H). Resting body temperature was similar between groups (36.7 ± 0.5 vs. 36.7 ± 0.1°C; 95% CI: 35.7–37.7 vs. 36.4–37.0, *P* = 0.9357; **Figure 4**).

**Figure 3:**
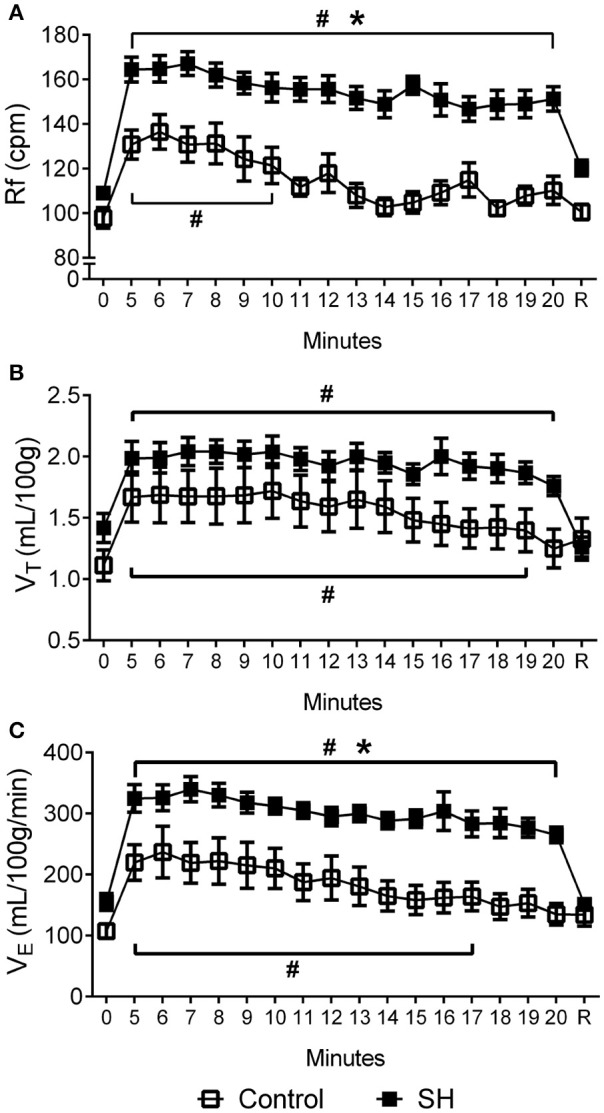
Hypoxic ventilatory response in rats exposed to 24 h of sustained hypoxia. Values of respiratory frequency (Rf, **A**), tidal volume (V_T_, **B**) and minute ventilation (V_E_, **C**) of rats exposed to 24 h of sustained hypoxia (SH, *n* = 12) or maintained under normoxia (control, *n* = 11) before (time 0), during hypoxia (7% O_2_ during 20 min, from time 5 to 20) and after the return to normoxia (R). ^#^Different from respective baseline; *Different from control group. *P* < 0.05.

**Figure 4:**
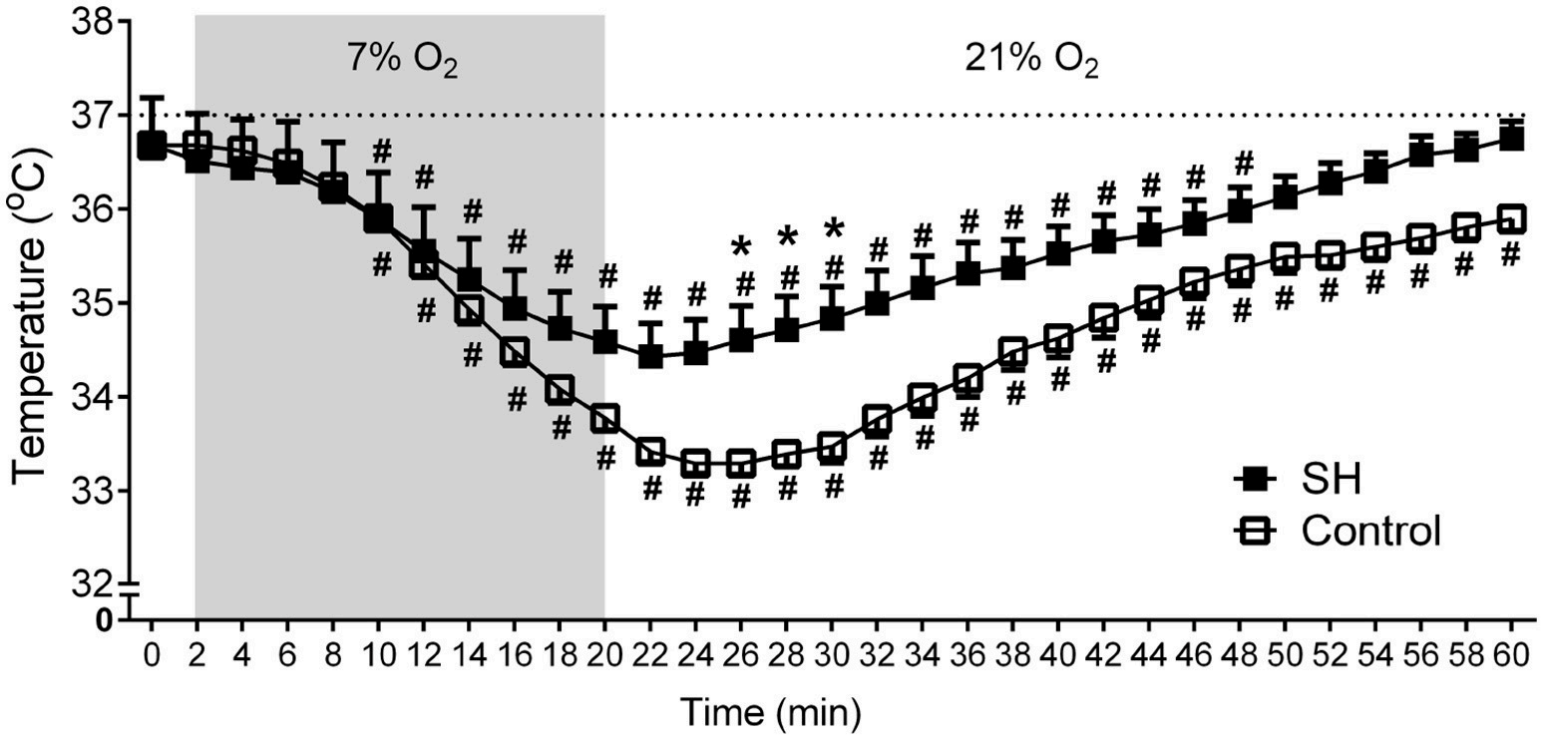
Baseline and hypoxia-induced changes in body temperature of rats exposed to 24 h of sustained hypoxia. Body temperature at normoxia (time 0), during hypoxia (7% O_2_, 20 min, gray area) and during the recovery period in rats exposed to 24 h of sustained hypoxia (SH, *n* = 12) or maintained under normoxia (control, *n* = 11). ^#^Different from respective baseline (time 0); *Different from control group. *P* < 0.05.

During a new episode of hypoxia (7% O_2_), both groups exhibited significant increases in Rf [*F*_(17, 340)_ = 13.93, *P* < 0.0001], V_T_ [*F*_(17, 357)_ = 23.03, *P* < 0.0001] and V_E_ [*F*_(17, 357)_ = 26.55, *P* < 0.0001], as demonstrated in the Figure 1. However, the magnitudes of increase in Rf [*F*_(1, 21)_ = 45.11, *P* < 0.001; 167 ± 5 vs. 137 ± 8 cpm, maximal increases at 7 and 6 min respectively; Figure 3A] and VE [*F*_(1, 21)_ = 13.99, *P* = 0.0013; 339.9 ± 20.6 vs. 236.7 ± 42.2 ml. 100 g^−1^.min^−1^; maximal increases at 7 and 6 min respectively; Figure 3C], but not in VT [*F*_(1, 21)_ = 3.215, *P* = 0.0881; 2.04 ± 0.11 vs. 1.67 ± 0.21 mL.100 g^−1^, maximal increases at 7 min; Figure 3B], were higher in SH than in the controls rats. With respect to body temperature, hypoxia caused a significant fall in both experimental groups [*F*_(30, 630)_ = 104,4, *P* < 0.0001, Figure 4). However, the reduction in body temperature was significantly smaller in SH than in the control group [*F*_(1, 21)_ = 4,359, *P* = 0.0492; 33 ± 0.1 vs. 34 ± 0.3°C; lower values at 22 and 26 min, respectively; Figure 4).

### Respiratory, sympathetic and carotid body afferent activities in *in situ* preparations of SH rats

**Figure 5:**
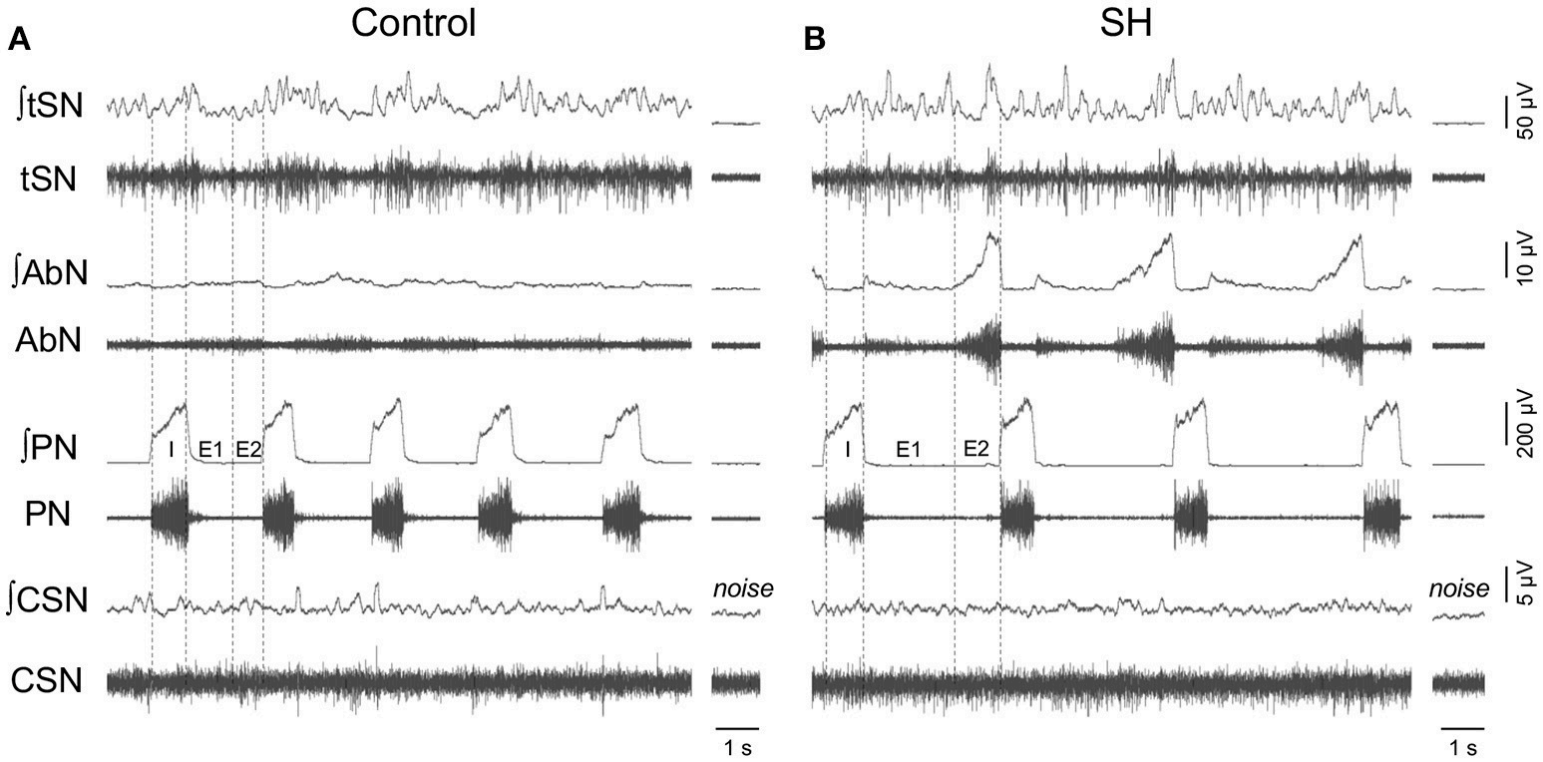
Baseline respiratory pattern, sympathetic and carotid sinus nerve activity of rats exposed to 24 h of sustained hypoxia. Raw and integrated (∫) recordings of abdominal (AbN), thoracic sympathetic (tSN), phrenic (PN), and carotid sinus (CSN) nerve activities from representative *in situ* preparations of control **(A)** and sustained hypoxia-conditioned rats **(B)**. The noise levels of each nerve recording are represented next to the corresponding trace. Dotted lines delineate the inspiratory phase (I), stage 1 of expiration (E1), and stage 2 of expiration (E2).

**Figure 6:**
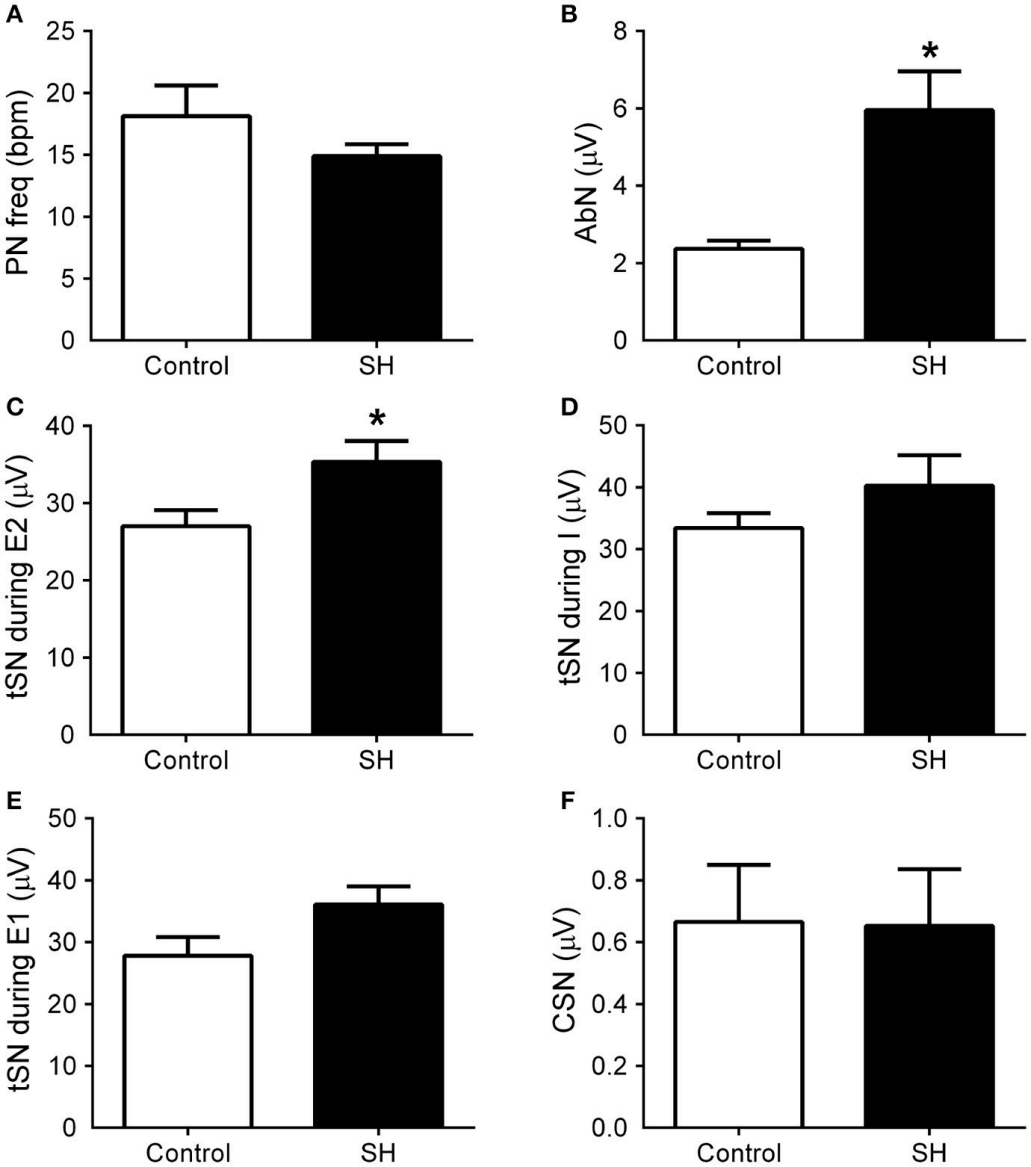
Baseline respiratory and sympathetic parameters of rats exposed to 24 h of sustained hypoxia. Average values of phrenic nerve frequency (PN, **A**), abdominal mean activity (AbN, **B**), thoracic sympathetic nerve activity (tSN) during E2 **(C)**, inspiratory **(D)** and E1 phases **(E)**, and carotid sinus nerve activity (CSN, **F**) of rats exposed to 24 h of sustained hypoxia (SH, *n* = 9) or maintained under normoxia (control, *n* = 8). *Different from control group, *P* < 0.05.

Under baseline conditions (hyperoxia/normocapnia), the respiratory pattern of *in situ* preparations of control rats (*n* = 8) was characterized by PN bursts in ramping pattern of discharge and low levels of AbN activity (Figure 5A). Also, tSN of control *in situ* preparations showed respiratory modulation with bursts of activity during the inspiratory/early-expiratory phase (Figure 5A) while CSN displayed very low activity. In the *in situ* preparations of SH rats (*n* = 9), the PN burst amplitude (197 ± 29 vs. 190 ± 20 μV, 95% CI: 130–263 vs. 143–236, *P* = 0.8515) and frequency (15 ± 1 vs. 18 ± 2 bpm, 95% CI: 13–17 vs. 12–24, *P* = 0.2230; Figure 6A) were similar to the control group. On the other hand, the AbN activity of SH rats showed novel bursts of discharge during late part of expiratory period (Figure 5B), which elevated baseline AbN activity levels compared to controls (6.0 ± 1.0 vs. 2.4 ± 00.2 μV, 95% CI: 3.2–8.8 vs. 1.9–2.9, *P* = 0.0011; Figure 6B). The tSN of SH preparations showed additional bursts during expiratory phase, coincident to the emergence of the AbN bursts (Figure 5B). Because of these additional expiratory bursts, tSN levels during E2 phase (35.3 ± 2.7 vs. 26.5 ± 2.3 μV, 95% CI: 29.1–41.6 vs. 22.1–31.9, *P* = 0.0296; Figure 6C), but not during inspiratory (40.3 ± 4.9 vs. 32.2 ± 2.4 μV, 95% CI: 28.9–51.6 vs. 27.7–39.1, *P* = 0.2488; Figure 6D) and E1 phases (36.1 ± 2.9 vs. 27.8 ± 3.0, 95% CI: 29.3–42.8 vs. 20.4–35.2, *P* = 0.0725 Figure 6E), were significantly higher in SH than in the control group. The average levels of baseline CSN activity of preparations of SH rats were similar to those observed in the control group (0.66 ± 0.18 vs. 0.67 ± 0.18 μV; 95% CI: 0.23–1.07 vs. 0.22–1.11, *P* = 0.9003; Figure 6F).

**Figure 7:**
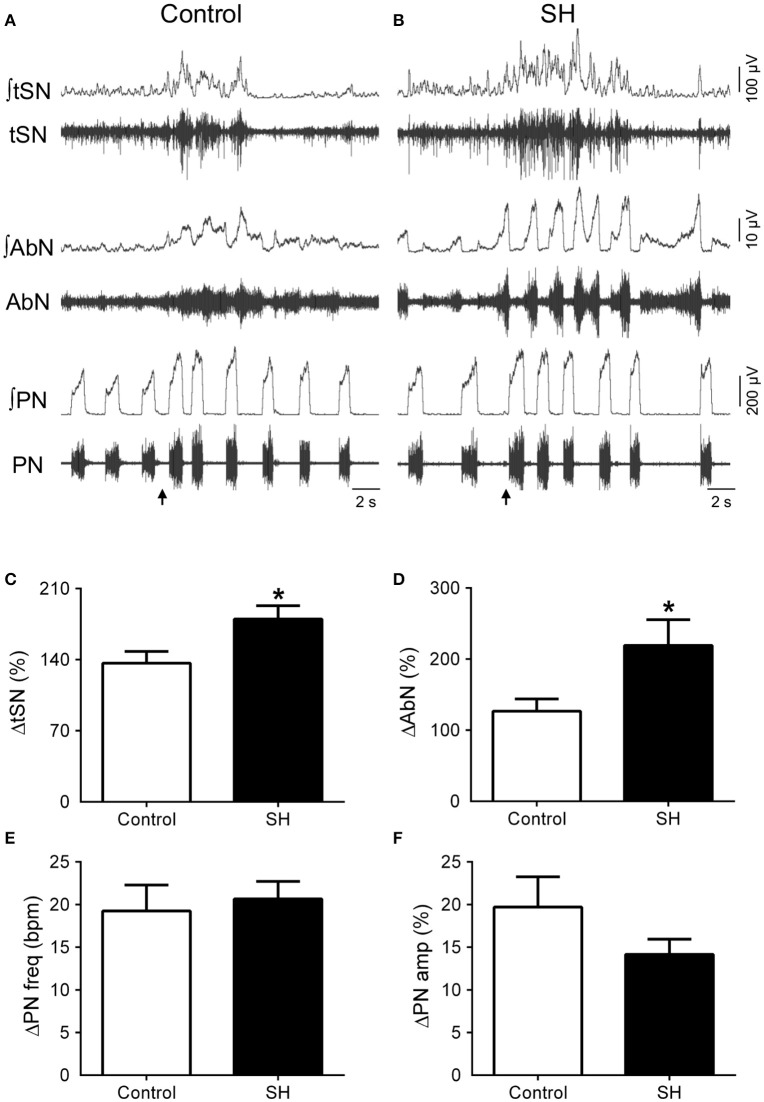
Respiratory and sympathetic responses to stimulation of peripheral chemoreceptors of rats exposed to 24 h of sustained hypoxia. Raw and integrated (∫) recordings of abdominal (AbN), thoracic sympathetic (tSN), phrenic nerve (PN) activities from representative control **(A)** and sustained hypoxia-conditioned *in situ* preparations **(B)**, illustrating the responses to peripheral chemoreceptor activation with KCN (0.05%, 50 μL; arrows). **(C–F)** Average changes in tSN, AbN, PN burst frequency and amplitude, respectively, of rats exposed to 24 h of sustained hypoxia (SH, *n* = 9) or maintained under normoxia (control, *n* = 8). *Different from control group, *P* < 0.05.

**Figure 8:**
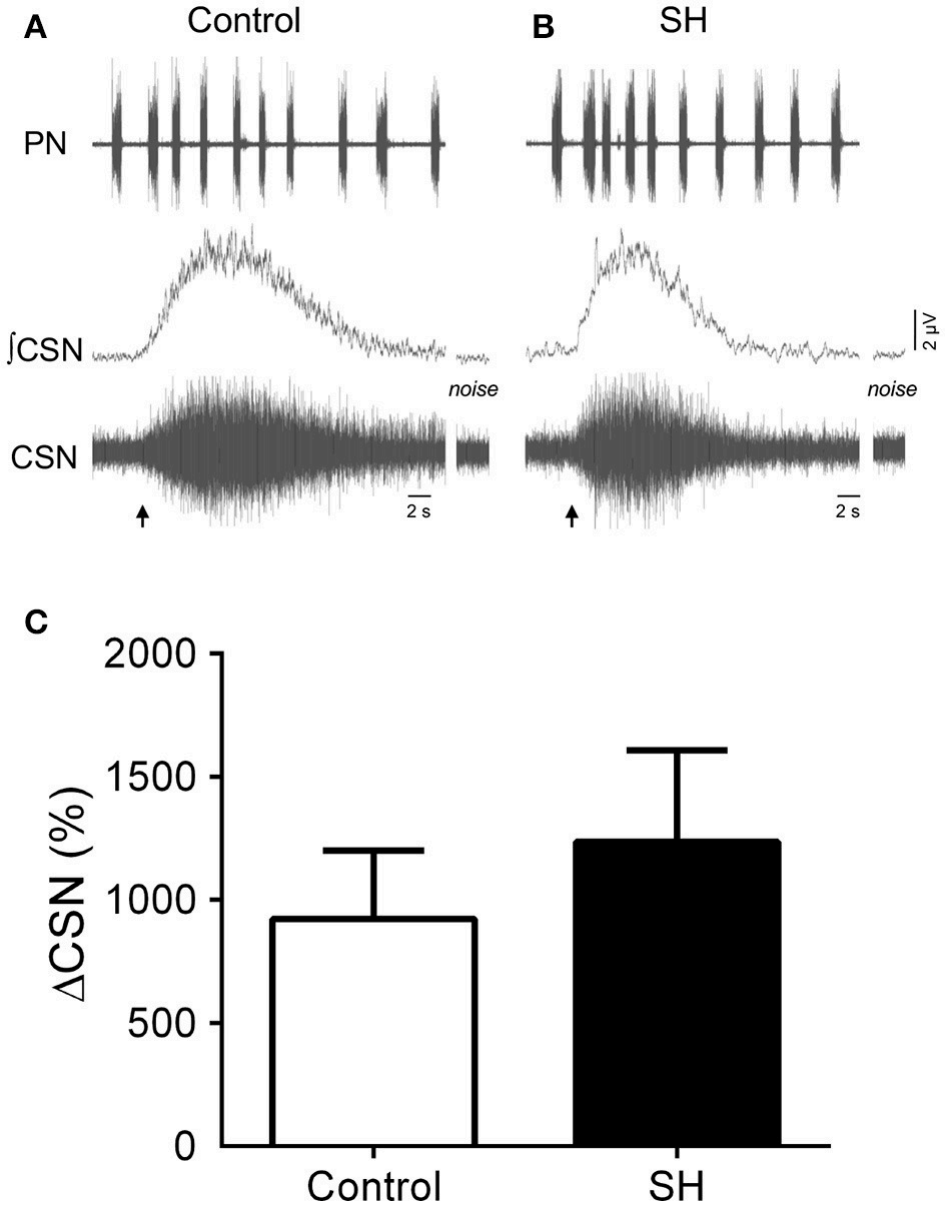
Sensory response of carotid body chemoreceptors of rats exposed to 24 h of sustained hypoxia. **(A)** Raw and integrated (∫) recordings of phrenic (PN) and carotid sinus nerve (CSN) activities from representative preparations of control **(A)** and sustained hypoxia-conditioned rats **(B)**, illustrating the excitatory response to peripheral chemoreceptor activation with KCN (0.05%, 50 μL; arrow). The noise levels of the carotid sinus nerve recording are represented next to the corresponding trace. **(C)** Average values of CSN excitatory response to peripheral chemoreceptor activation in rats exposed to 24 h of sustained hypoxia (SH, *n* = 9) or maintained under normoxia (control, *n* = 8).

In response to stimulation of peripheral chemoreceptors with KCN, both groups of *in situ* preparations showed marked increases in PN, AbN, tSN, and CSN activities (Figures 7A,B, 8A,B). The magnitude of the tSN (ΔtSN: 180 ± 13 vs. 137 ± 12 %, 95% CI: 150–210 vs. 109–164; *P* = 0.0267; Figure 7C) and AbN excitatory responses (ΔAbN: 219 ± 36 vs. 127 vs. 17 %, 95% CI: 119–320 vs. 86–168, *P* = 0.0247; Figure 7D) were amplified in the SH group compared to controls. On the other hand, the responses of increase in PN burst frequency (ΔPN: 21 ± 2 vs. 19 ± 3 bpm; 95% CI: 16–25 vs. 12–26, *P* = 0.6985; Figure 7E) and amplitude (ΔPN: 13 ± 2 vs. 23 ± 5 %; 95% CI: 10–18 vs. 11–28, *P* = 0.1714; Figure 7F), and the CSN activity (ΔCSN: 1,235 ± 372 vs. 923 ± 278%, 95% CI: 376–2,094 vs. 242–1,603, *P* = 0.8832; Figure 8C) were similar between groups.

## Discussion

In the present study, we report for the first time that 24 h of SH (10% O_2_) is able to elevate baseline pulmonary ventilation under normoxic and normocapnic conditions, as well as to potentiate the hypoxic ventilatory response in juvenile rats. These findings obtained in unanesthetized animals parallel with our data from *in situ* preparations showing that short-term SH evokes novel expiratory bursts in abdominal (active expiration) and sympathetic activities at baseline conditions and amplifies respiratory and sympathetic reflex responses to peripheral chemoreceptor stimulation. Interestingly, baseline and evoked afferent activity of carotid body chemoreceptors of SH-conditioned rats were not different from control rats maintained at normoxia. Our data indicate that augmented resting ventilation, baseline sympathetic overactivity, and enhanced ventilatory responses to hypoxia in juvenile rats exposed to 24 h of SH are not critically dependent on increased basal and sensorial activity of carotid body chemoreceptors. Based upon that, our study provides novel functional data that support the possibility that adaptations in the central nervous system may precede the development of augmented carotid body chemosensory activity in rats exposed to chronic SH and contribute to the maintenance of VAH and to the amplified ventilatory and sympathetic responses to a new episode of hypoxia.

Accumulating evidence indicates that SH exposure promotes a progressive increase in baseline ventilation in mammals, which may persist for hours or days upon reoxygenation (Powell et al., 1998). Herein, we found that 24 h of SH was sufficient to elevate baseline ventilation in juvenile rats. Specifically, we observed that SH-conditioned rats exhibited higher respiratory frequency and tidal volume under normoxia/normocapnia, measured at least 1 h after the return to normoxia. On the basis of our *in situ* data, we speculate that this augmented resting ventilation of SH-conditioned rats results from changes in the respiratory pattern and the emergence of active expiration. The occurrence of high amplitude bursts in abdominal expiratory motor activity, as seen in the *in situ* preparations of SH rats under baseline conditions (hyperoxia and normocapnia), are suggested to facilitate breathing by accelerating the expiratory flow, recruiting the expiratory reserve volume, or improving the length-tension relationship of the diaphragm (Jenkin and Milsom, 2014). Also, the active expiration pattern is associated with decreases in the upper airway resistance due to modifications in the abductor and adductor control of laryngeal muscles (Moraes et al., 2014). In agreement with these observations, we found that SH rats showed reduced time of expiration, augmented V_T_/T_i_ ratio (indicating that the lung filled more rapidly) and elevated peak inspiratory and expiratory flows. The *in situ* preparations of SH rats did not exhibit significant changes in respiratory frequency as seen *in vivo*, which may be related to the decortication or to the absence of some peripheral feedback inputs, mainly from pulmonary stretch receptors (Zoccal et al., 2009), which contribute to breathing frequency control (Mörschel and Dutschmann, 2009; Lemes and Zoccal, 2014). In spite of that, we suggest that the generation of active expiratory pattern and the recruitment of abdominal expiratory activity is an important mechanism underpinning the VAH, at least to short-term SH.

Previously, Moraes et al. (2014) reported that the abdominal hyperactivity after the exposure to SH for 24 h is linked to the activation of expiratory neurons of the pFRG. The pFRG is a conditional expiratory oscillator that is synaptically suppressed at resting conditions (normoxia/normocapnia) but emerges in situations of elevated ventilatory demand (hypoxia and hypercapnia) to provide the excitatory drive necessary for the generation of expiratory bursts in the abdominal motor activity (Abdala et al., 2009; Pagliardini et al., 2011; Lemes et al., 2016a). The pFRG neurons are also suggested as an important source of excitatory inputs to pre-sympathetic neurons of rostral ventrolateral medulla, producing expiratory-related bursts in sympathetic outflow in association with active expiration (Molkov et al., 2011). Acute activation of peripheral chemoreceptors was found to stimulate the expiratory neurons of the pFRG (Moraes et al., 2012). Considering that carotid body chemoreceptors are required for the development of VAH after SH (Smith et al., 1986) and that carotid body chemosensory sensitization occurs during early stages of exposure to chronic SH, at least in goats and cats (Powell et al., 1998; Bisgard, 2000), we hypothesized that carotid body chemoreceptor activity would be enhanced after 24 h of SH, contributing to the generation of active expiration. We found that SH rats exhibited amplified ventilatory responses to hypoxia *in vivo*, as well as enhanced abdominal and sympathetic responses to KCN *in situ*. However, baseline CSN activity was similar between groups, indicating that the hyperactivation of pFRG neurons after the short-term SH mainly depends on central mechanisms. Moreover, the magnitude of chemosensory response to KCN was not significantly different between SH and control groups. These data indicate that the chemosensory activity of carotid body is not critically altered after 24 h of SH, and the magnified respiratory and sympathetic chemoreflex responses, at this stage, may be mainly centrally mediated. Although not statistically different, the SH group showed a trend of increase (~30%) in the average CSN response to KCN. We interpret this non-significant increase in the KCN-evoked response of SH-conditioned rats as a possible sensorial potentiation that is under development, as reported in rats exposed to longer periods of hypoxia (Barnard et al., 1987; Bisgard, 2000). Moreover, by the fact that the sensory responses were tested using KCN, we may have only evaluated the maximal responses of carotid body chemoreceptors. Therefore, our data do not exclude the possibility that 24 h of SH may modify the sensory response to weaker hypoxic stimulus.

Functional changes have been reported in brainstem regions related to the processing of the peripheral chemoreceptor inputs. At the level of the nucleus of solitary tract (NTS), the first synaptic station of carotid body chemoreceptor afferents (Mifflin, 1992), the intrinsic excitability and excitatory synaptic transmission on 2nd-order neurons was found enhanced after 24 h of SH (Accorsi-Mendonça et al., 2015). Also at the level of the NTS, there is also evidence of plasticity of glutamatergic neurotransmission (Pamenter et al., 2014) and activation of microglia and astrocytes contributing to VAH to chronic SH (Tadmouri et al., 2014; Stokes et al., 2017). Within the ventral respiratory column, specifically at the level of pre-Bötzinger complex (pre-BötC), it has been suggested the existence of cells that are sensitive to O_2_ and play a role in the hypoxic ventilatory response (Solomon et al., 2000; Angelova et al., 2015). These central respiratory O_2_ sensors might undergo plasticity after SH exposure and play a role in VAH and augmented ventilatory response to hypoxia. Modifications in the activity of expiratory neurons of the Bötzinger complex (BötC), located in the ventral respiratory column, were also documented in rats after 24 h of SH (Moraes et al., 2014). The NTS, pre-BötC and BötC establish synaptic interactions with the pFRG region (Rosin et al., 2006) and, therefore, may constitute potential mechanisms contributing to the hyperactivation of expiratory neurons after SH exposure. In the pFRG, we previously identified that activation of serotoninergic receptors during intermittent hypoxia promotes long-lasting activation of expiratory neurons and the emergence of abdominal expiratory bursts at resting conditions (Lemes et al., 2016b). These findings allow us to speculate that the activation of local neuromodulatory mechanisms in the pFRG may also play a role in the development of active expiration and VAH during short-term SH.

Although acute hypoxia reduces body temperature (Mortola, 2016), we verified that SH for 24 h did not alter body temperature of rats at baseline conditions. Interestingly, we verified that the hypoxia-induced fall in body temperature was smaller in SH-conditioned rats. The hypothermic response to acute hypoxia seems to be mainly dependent on reductions in the thermogenesis, such as reductions in shivering (Barros et al., 2001) or inhibition of the sympathetic activity to the brown adipose tissue (Madden and Morrison, 2005). Taking into consideration the latter mechanism, we verified that SH-conditioned rats presented higher levels of sympathetic activity under baseline conditions and during peripheral chemoreceptor stimulations. We previously demonstrated that the sympathetic overactivity after 24 h of SH contributes to elevate baseline arterial pressure levels (Moraes et al., 2014). Herein, we also consider that the enhanced baseline sympathetic activity after SH may have functional significance to thermogenesis regulation, contributing to maintain body temperature in physiological levels at baseline conditions as well as preventing greater decreases during a new hypoxia challenge. Apparently, based on our recordings from carotid sinus activity, the attenuated fall in body temperature of SH rats in response to hypoxia were not dependent on changes in the peripheral chemoreceptor activity. Therefore, it is also possible that SH exposure may have also elicited changes in the central thermosensitivity mechanisms, such as those located in the hypothalamus (Tattersall and Milsom, 2009), and modified the thermogenic response to hypoxia. All hypotheses still require additional experiments to be proven.

In conclusion, our study provides evidence in favor to the notion that the maintenance of VAH and sympathetic overactivity of juvenile rats exposed to 24 h of SH are not linked to changes on baseline and chemosensory activity of carotid body chemoreceptors. In this regard, we suggest that functional adaptations in central mechanisms related to the control of respiratory pattern and/or the gain of peripheral chemoreflex may primarily contribute to the respiratory and sympathetic changes observed during the initial stages of acclimatization to SH in juvenile rats. After longer periods of exposure (more than 24 h), increased peripheral chemoreceptor afferent activity emerges (Barnard et al., 1987; Bisgard, 2000) and further contributes to the progression and maintenance of cardiorespiratory adaptations to chronic SH. Therefore, the identification of the cellular, synaptic and circuitry-based alterations induced by SH is important to fully understand the mechanisms underpinning the development of respiratory and sympathetic adaptation/maladaptation observed in subjects sojourning in high altitudes as well as in pathological states associated with chronic hypoxia exposure, such as chronic obstructive pulmonary disease and chronic heart failure (Dempsey and Smith, 2014).

## Author contributions

DZ, GP, EC, KF, ES, and MM: designed the experimental protocols; KF, and MM: performed the *in vivo* experiments; ES: performed the *in situ* experiments; DZ, KF, MM, and ES: analyzed the data; DZ, KF, ES, MM, GP, and EC: interpreted the data, drafted, revised, and approved the final version of the manuscript.

### Conflict of interest statement

The authors declare that the research was conducted in the absence of any commercial or financial relationships that could be construed as a potential conflict of interest.
